# Role of Membrane Microdomains in Compartmentation of cAMP Signaling

**DOI:** 10.1371/journal.pone.0095835

**Published:** 2014-04-21

**Authors:** Shailesh R. Agarwal, Pei-Chi Yang, Monica Rice, Cherie A. Singer, Viacheslav O. Nikolaev, Martin J. Lohse, Colleen E. Clancy, Robert D. Harvey

**Affiliations:** 1 Department of Pharmacology, University of Nevada School of Medicine, Reno, Nevada, United States of America; 2 Department of Pharmacology, University of California Davis, Davis, California, United States of America; 3 European Heart Research Institute Gottingen, University of Göttingen, Göttingen, Germany; 4 Department of Pharmacology, University of Würzburg, Würzburg, Germany; Tohoku University, Japan

## Abstract

Spatially restricting cAMP production to discrete subcellular locations permits selective regulation of specific functional responses. But exactly where and how cAMP signaling is confined is not fully understood. Different receptors and adenylyl cyclase isoforms responsible for cAMP production are not uniformly distributed between lipid raft and non-lipid raft domains of the plasma membrane. We sought to determine the role that these membrane domains play in organizing cAMP responses in HEK293 cells. The freely diffusible FRET-based biosensor Epac2-camps was used to measure global cAMP responses, while versions of the probe targeted to lipid raft (Epac2-MyrPalm) and non-raft (Epac2-CAAX) domains were used to monitor local cAMP production near the plasma membrane. Disruption of lipid rafts by cholesterol depletion selectively altered cAMP responses produced by raft-associated receptors. The results indicate that receptors associated with lipid raft as well as non-lipid raft domains can contribute to global cAMP responses. In addition, basal cAMP activity was found to be significantly higher in non-raft domains. This was supported by the fact that pharmacologic inhibition of adenylyl cyclase activity reduced basal cAMP activity detected by Epac2-CAAX but not Epac2-MyrPalm or Epac2-camps. Responses detected by Epac2-CAAX were also more sensitive to direct stimulation of adenylyl cyclase activity, but less sensitive to inhibition of phosphodiesterase activity. Quantitative modeling was used to demonstrate that differences in adenylyl cyclase and phosphodiesterase activities are necessary but not sufficient to explain compartmentation of cAMP associated with different microdomains of the plasma membrane.

## Introduction

Many different G protein coupled receptors (GPCRs) are capable of stimulating cAMP production. Furthermore, this ubiquitous second messenger can regulate a variety of cellular activities. Yet despite the fact that multiple receptors can stimulate cAMP production in any given cell, they do not always produce identical functional responses. Such observations led to the original hypothesis that that receptor activation does not necessarily produce a uniform increase in cAMP throughout the cell [1], [2]. Localized increases in cAMP allow for targeted regulation of cAMP-dependent responses in distinct subcellular compartments.

Early studies investigating compartmentalized cAMP signaling focused on differences in second messenger production associated with membrane and non-membrane fractions of cells [1]–[4]. This was due to technical limitations that only allowed cAMP measurements in particulate (membrane) or supernatant (cytosolic) fractions of cell or tissue homogenates. More recently the development of various biosensors has made it possible to measure changes in cAMP activity in intact living cells [5]–[7]. However, most studies have still focused on differences between cAMP activity near the plasma membrane and the bulk cytoplasmic compartment [8]–[12]. The results suggest that receptor activation produces differences in the magnitude and the time course of cAMP responses observed in these two compartments. However, the assumption has often been that cAMP signaling near the plasma membrane is uniform.

A number of factors may actually contribute to non-uniformity of cAMP signaling in different subcellular compartments. Localized differences in cAMP metabolism by phosphodiesterases (PDEs) are often cited [9], [10], [12]–[15]. However, inhomogeneities in the distribution of receptors and other signaling proteins responsible for cAMP production are also believed to play a key role [16], [17]. Even though many of these proteins are associated with the plasma membrane, there is clear evidence not all are distributed homogenously. Many are either included or excluded from lipid rafts, which are detergent-resistant membrane domains rich in cholesterol. These regions of the membrane, which in some cells include caveolae, provide a platform for the aggregation of various signaling proteins through lipid-protein and protein-protein interactions [18]–[21].

Examples of receptors that exhibit non-uniform distribution between lipid raft and non-lipid raft domains of the plasma membrane include β-adrenergic receptors (βARs) and E type prostaglandin receptors (EPRs). Both are capable of stimulating cAMP production, yet βARs are often associated with the cholesterol-rich, buoyant fractions of the plasma membrane, as identified by sucrose density centrifugation, while EPRs are only found in non-raft fractions [8], [16], [22]–[27]. Likewise, there are also differences in the distribution of various isoforms of adenylyl cyclase, the enzyme responsible for synthesis of cAMP, between raft and non-raft membrane fractions [16], [17], [26]–[28]. These observations raise the possibility that cAMP production near the plasma membrane is not uniform. The purpose of this study was to test this hypothesis using FRET-based biosensors targeted to lipid raft and non-lipid raft microdomains of the plasma membrane. The results demonstrate that cAMP signaling associated with the plasma membrane is not homogeneous and that there are differences between raft and non-raft domains that are due to variations in the distribution of receptors as well as adenylyl cyclase activity.

## Results

### Membrane Microdomain Targeting of FRET-based Biosensors

**Figure 1:**
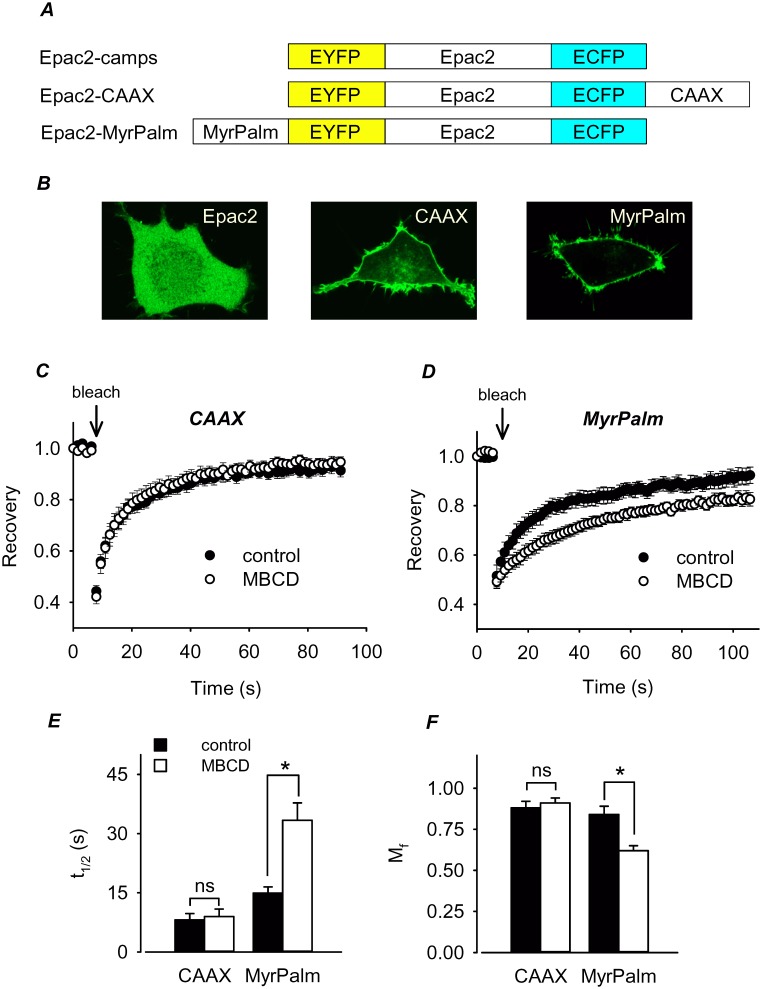
FRET-based biosensors targeted to different membrane microdomains. ***A*** *,* structure of individual Epac2-based biosensor constructs. ***B*** *,* Confocal images of HEK293 cells expressing Epac2-camps (Epac2), Epac2-CAAX (CAAX), and Epac2-MyrPalm (MyrPalm). ***C*** and ***D***, effect of cholesterol depletion on the time course of fluorescence recovery after photobleaching in HEK293 cells expressing Epac2-CAAX or Epac2-MyrPalm. ***E*** and ***F***, summary of fluorescence recovery half-time (t_1/2_) and mobile fraction (M_f_) in control and MBCD-treated cells. Differences between control (n = 14) and MBCD-treated (n = 10) cells expressing Epac2-CAAX were not significant (t_1/2_, p = 0.378; M_f_, p = 0.517). Differences between control (n = 5) and MBCD-treated (n = 8) cells expressing Epac2-MyrPalm were significant (t_1/2_, p = 0.002; M_f_, p = 0.003).

Epac2-camps is a freely diffusible FRET-based biosensor that responds to changes in cAMP occurring throughout the cytosolic compartment of cells [29], [30]. Epac2-CAAX was targeted to non-lipid raft domains of the plasma membrane, while Epac2-MyrPalm was targeted to lipid rafts. Previous studies have demonstrated that the targeting sequences used here direct expression to these specific membrane domains [31]–[34]. Confocal imaging confirmed that Epac2-camps is expressed throughout the cytoplasm, while Epac2-CAAX and Epac2-MyrPalm are concentrated at the plasma membrane (**figure 1**). However, lipid raft and non-lipid raft membrane domains cannot be resolved using conventional microscopy techniques [35], [36]. Therefore, to verify that Epac2-MyrPalm was being targeted to lipid rafts, while Epac2-CAAX was not, we measured the mobility of each using FRAP in control cells and cells in which lipid rafts had been disrupted by depleting membrane cholesterol with methyl-β-cyclodextrin (MBCD). Disrupting lipid rafts in this manner is expected to have a greater effect on the mobility of proteins associated specifically with lipid rafts.

The results demonstrate that cholesterol depletion did not significantly affect the mobility of Epac2-CAAX (**figures 1C, 1E, and 1F**). In control cells, the fluorescence recovery half time (t_1/2_) and mobile fraction (M_f_) were 8.1±1.6 s and 0.88±0.040 (n = 14), respectively. The corresponding values in MBCD-treated cells were 9.0±1.9 s and 0.91±0.030 (n = 10), respectively. In contrast, cholesterol depletion did significantly alter the mobility of Epac2-MyrPalm (**figure 1D, 1E, and 1F**). In control cells the t_1/2_ and M_f_ were 15±1.6 s and 0.84±0.050 (n = 5), respectively, while in MBCD-treated cells the t_1/2_ increased to 33±4.4 s and the M_f_ decreased to 0.62±0.030 (n = 8). The decrease in mobility is consistent with the effect that cholesterol depletion has been shown to have on raft associated proteins in other studies [37]–[41]. These results support the idea that Epac2-MyrPalm was targeted to lipid raft domains, while Epac2-CAAX was not.

### Receptor-dependent cAMP Production in Different Membrane Domains

Having obtained evidence that the two membrane bound probes are targeted to different microdomains in the plasma membrane, we then looked at the cAMP responses occurring in these locations and compared them to the response observed throughout the entire cytoplasmic compartment using Epac2-camps. We started by measuring responses to endogenous βAR and EPR stimulation. In HEK293 cells, like many other cell types, βARs have been shown to be enriched in lipid raft domains while EPRs are excluded from those membrane fractions [8].

**Figure 2:**
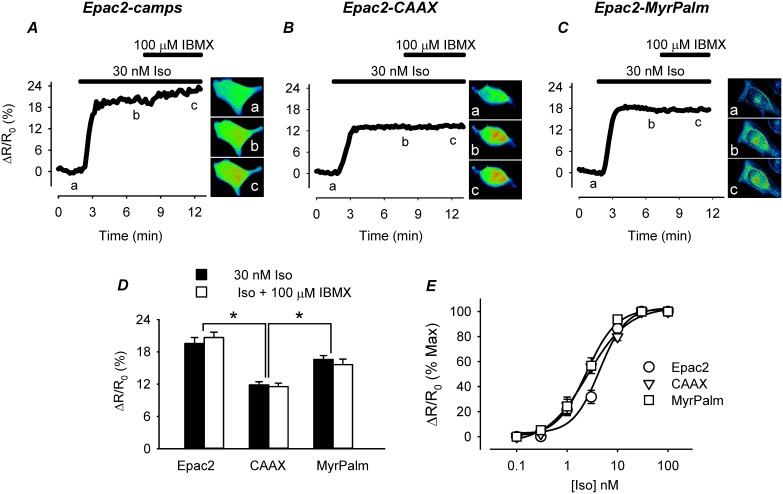
Effect of the β-adrenergic receptor (βAR) agonist isoproterenol (Iso) on cAMP responses detected by biosensors targeted to different microdomains. ***A***-***C***, time course of changes in the FRET response (ΔR/R_0_) and corresponding pseudocolor images obtained from cells expressing Epac2-camps (Epac2), Epac2-CAAX (CAAX), and Epac2-MyrPalm (MyrPalm), under control conditions (*a*), and following exposure to 30 nM Iso (*b*) and 30 nM Iso+100 µM IBMX (*c*). ***D***, comparison of average FRET responses to maximal βAR stimulation (30 nM Iso) and maximal cAMP production (Iso+100 µM IBMX). (n = 5–9; *p<0.001) ***E***, concentration-response curves for Iso activation of FRET response in cells expressing different biosensors (n = 3–11). Concentrations of Iso producing half maximal activation (EC_50_) of Epac2-camps (4.3±1.0 nM), Epac2-CAAX (2.5±0.37 nM), and Epac2-MyrPalm (2.4±0.20 nM) were not significantly different (p>0.05).

All three probes responded when cells were exposed to the βAR agonist isoproterenol (Iso) (**figure 2**). The FRET responses (ΔR/R_0_) of Epac2-camps (20±1.1%, n = 8) and Epac2-MyrPalm (17±0.72%, n = 9) were similar in magnitude. However, the size of the response detected by the Epac2-CAAX probe (12±0.58%, n = 5) in non-lipid raft domains was significantly smaller (**figure 2D**). Previous studies have suggested that differences in the size of cAMP responses detected using targeted biosensors similar to the ones used in the present study can be attributed to differences in subcellular PDE activity [9]. However, addition of the non-specific PDE inhibitor 3-isobutyl-1-methylxanthine (IBMX) did not alter the magnitude of the maximal Iso response detected by any of the probes.

**Figure 3:**
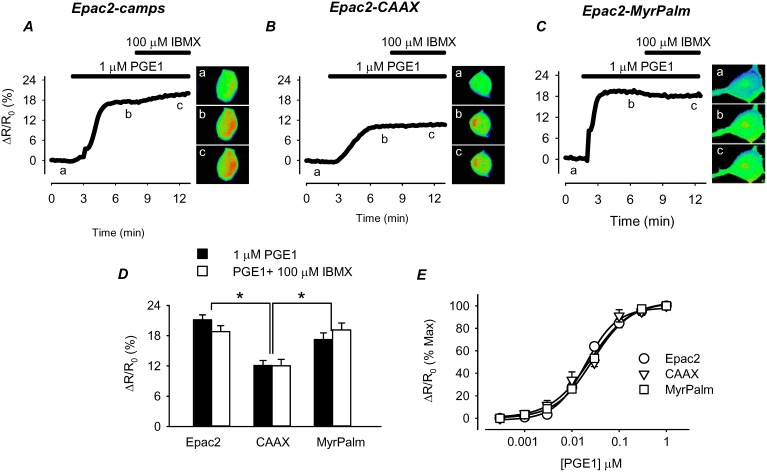
Effect of the E type-prostaglandin receptor (EPR) agonist PGE1 on cAMP responses detected by biosensors targeted to different microdomains. ***A–C*,** time course of changes in FRET response (ΔR/R_0_) and corresponding pseudocolor images from cells expressing Epac2-camps (Epac2), Epac2-CAAX (CAAX), and Epac2-MyrPalm (MyrPalm), under control conditions (*a*), and following exposure to 1 µM PGE1 (*b*) and 1 µM PGE1+100 µM IBMX (*c*). ***D***, comparison of average FRET responses to maximal EPR stimulation (1 µM PGE1) and maximal cAMP production (PGE1+100 µM IBMX). (n = 4; *p<0.001) ***E***, concentration-response curves for PGE1 activation of FRET response in cells expressing the different biosensors (n = 4–11). Concentrations of PGE1 producing half maximal activation (EC_50_) of Epac2-camps (20±1.7 nM), Epac2-CAAX (25±6.0 nM), and Epac2-MyrPalm (28±1.4 nM) were not significantly different (p>0.05).

Another possible explanation for the smaller size of the response detected by Epac2-CAAX is that there are fewer βARs found in non-lipid raft regions of the plasma membrane [8]. To evaluate this possibility, we next examined the responses to activation of EPRs, using prostaglandin E1 (PGE1) as an agonist (**figure 3**). Because EPRs are absent from lipid-raft domains, we predicted that the pattern of responses detected in different subcellular locations might differ from that observed following βAR activation. However, this was not the case. The magnitude of the maximal FRET response produced by PGE1 was not significantly different from that produced by Iso. Following exposure to a maximally stimulating concentration of PGE1, the FRET responses produced by Epac2-camps (21±0.97%, n = 23) and Epac2-MyrPalm (17±1.3, n = 15) were significantly larger than the response produced by Epac2-CAAX (12±1.0, n = 20) (**figure 3D**). Again, this difference could not be attributed to PDE activity, since addition of IBMX had no effect on the magnitude of the response produced by any of the probes. These results indicate that maximal activation of both β-adrenergic and E-type prostaglandin receptors produces cAMP responses that saturate all three probes.

**Figure 4:**
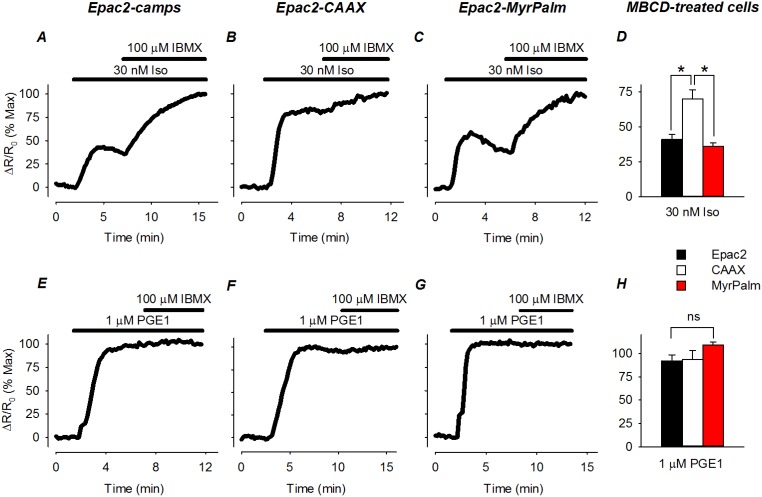
Effect of cholesterol depletion on the sensitivity of receptor-dependent cAMP responses detected by biosensors targeted to different microdomains. ***A*–*C*** *,* time course of changes in normalized FRET response (ΔR/R_0_) in cells expressing Epac2-camps (Epac2), Epac2-CAAX (CAAX), and Epac2-MyrPalm (MyrPalm) following exposure to a maximally stimulating concentration of the βAR agonist isoproterenol (Iso). Responses are normalized to the magnitude of the maximal FRET response produced by Iso plus IBMX. ***D***, comparison of average changes in normalized FRET responses to 30 nM Iso (n = 6–10, *p<0.001). ***E–G***, time course of changes in normalized FRET response to a maximally stimulating concentration of the EPR agonist PGE1. Responses are normalized to the magnitude of the maximal FRET response produced by exposure to PGE1 plus IBMX. ***H***, comparison of average changes in normalized FRET responses to 1 µM PGE1 (n = 4; ns  =  not significant).

The lack of any obvious difference between responses produced by Iso and PGE1 might be explained if the receptors involved are not actually associated with different microdomains in the plasma membrane. To evaluate this possibility, we examined the responses produced by Iso and PGE1 in cells pretreated with MBCD. Cholesterol depletion has been shown to disrupt lipid rafts and selectively alter responses to receptors found in those membrane domains [42]. In MBCD-treated cells, we found that the magnitude of the responses produced by βAR stimulation were smaller in all three subcellular locations (**figure 4**). This was demonstrated by normalizing the size of the FRET response produced by a maximally stimulating concentration of Iso to that observed following maximal activation of the probe by exposure to Iso plus IBMX. In control cells, the size of the FRET response observed in the presence of Iso plus IBMX was the same as the size of the response to Iso alone (see **figure 2**). However, in MBCD-treated cells, addition of IBMX resulted in a significant increase in the size of the FRET response produced by maximal βAR stimulation alone. This indicates that the sensitivity of the cAMP response to βAR stimulation had been reduced. However, the size of the normalized FRET responses of Epac2-camps (41±3.8% of max; n = 6) and Epac2-MyrPalm (36±2.6% of max; n = 10) were significantly smaller than the FRET response of Epac2-CAAX (70±6.5% of max; n = 8) in non-lipid raft domains (**figure 4D**). The change in the relative size of the response to Iso was not due a change in the size of the responses to Iso plus IBMX, since they were the same as in control cells. The fact that cholesterol depletion affected the Epac2-camps response supports the idea that βARs found in lipid raft domains contribute to global cAMP responses. The observation that there was an Epac2-CAAX response that was significantly less sensitive to cholesterol depletion suggests that there are at least some βARs associated with non-lipid raft regions of the plasma membrane.

Unlike the effects it had on βAR mediated responses, cholesterol depletion had no detectible effect on the responses to EPR activation (**figures 4E–H**). Just as in control cells, PGE1 elicited FRET responses from all three probes that were not significantly different from the maximal response observed following addition of IBMX: Epac2-camps, 92±6.5% of max (n = 4); Epac2-MyrPalm, 110±3.0% of max (n = 4); and Epac2-CAAX, 94±9.4% of max (n = 4). This is consistent with the idea that cAMP produced by exposure to PGE1 is due to activation of EPRs found in non-lipid raft fractions of the plasma membrane.

### Basal cAMP Levels in Different Membrane Domains

The results described above support the idea that the membrane domain in which βARs are found can determine whether or not they produce local or global cAMP responses in HEK293 cells. However, this does not explain why the response of Epac2-CAAX, which is associated with non-lipid raft regions of the plasma membrane, is significantly smaller than the responses associated with either the bulk cytoplasmic domain or lipid raft domains in control cells (see **figures 2** **and** **3**). An alternative explanation is that basal levels of cAMP vary in different subcellular locations within the cell. It is often assumed that basal levels of cAMP throughout the cell are below the threshold for detection by biosensors. But what if basal cAMP levels in cytoplasmic regions associated with non-lipid rafts are actually high enough to partially activate the Epac2-CAAX sensor? Subsequent stimulation of cAMP production would elicit a maximal FRET response (due to saturation of the biosensor) that appears smaller than cAMP responses in other microdomains.

**Figure 5:**
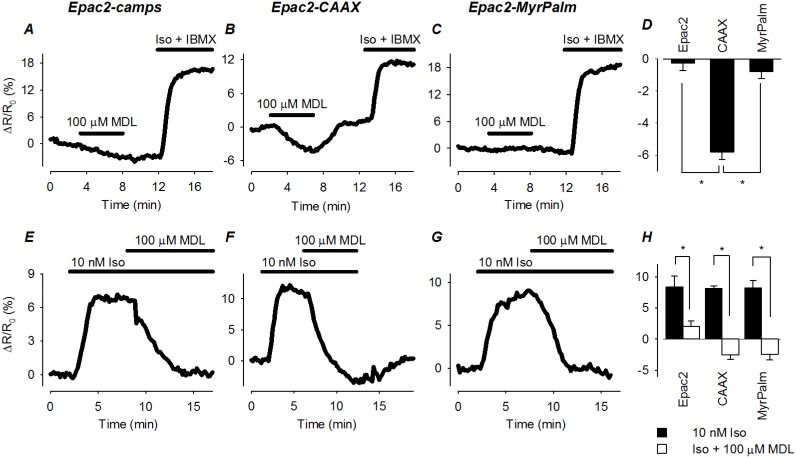
Effect of direct adenylyl cyclase (AC) inhibition on basal and β-adrenergic receptor (βAR) stimulated cAMP activity in different microdomains. ***A–C***, time course of changes in FRET response (ΔR/R_0_) in cells expressing Epac2-camps (Epac2), Epac2-CAAX (CAAX), and Epac2-MyrPalm (MyrPalm) following exposure to the AC inhibitor MDL12330A (MDL; 100 µM). Subsequent treatment with isoproterenol (Iso; 30 nM) plus IBMX (100 µM) was used to elicit a maximal FRET response. ***D***, comparison of average changes in FRET responses (*p<0.001). ***F–G***, time course of changes in FRET response (ΔR/R_0_) in cells expressing Epac2-camps (Epac2), Epac2-CAAX (CAAX), and Epac2-MyrPalm (MyrPalm) following exposure to the adenylyl cyclase inhibitor MDL12330A (MDL; 100 µM) after first stimulating cAMP production with the βAR agonist isoproterenol (Iso; 10 nM). ***H***, comparison of average changes in FRET responses to 10 nM Iso and Iso+100 µM MDL12330A (n = 4–11; *p<0.05).

The idea that basal cAMP concentrations vary in different subcellular compartments has been demonstrated in other cells types [28], [30]. To determine if this might also be the case in HEK293 cells, we used the AC inhibitor MDL12330A (MDL) [43]. Upon exposure to 100 µM MDL, there was no obvious change in the FRET response of Epac2-camps (−0.26±0.45; n = 9) and Epac2-MyrPalm (−0.78±0.43; n = 8). However, MDL did produce a significant decrease in the FRET response of Epac2-CAAX (−5.8±0.45; n = 15) that was reversible upon washout (**figures 5A–D**), suggesting that basal cAMP levels are significantly higher in non-lipid raft regions of the cell. To rule out the possibility that MDL might be affecting the Epac2-CAAX probe in a non-specific manner, we also treated cells expressing the different biosensors with MDL after first stimulating cAMP production with Iso. Under these conditions, exposure to MDL inhibited the FRET response of all three biosensors (**figures 5E–H**). These data indicate that the effects of MDL are due to inhibition of AC activity, supporting the idea that basal levels of cAMP associated with non-lipid raft regions of the plasma membrane are indeed higher than subcellular regions associated with lipid rafts and the bulk cytoplasmic domain.

Comparing the difference between the minimum response observed following exposure to 100 µM MDL and the maximum response observed following exposure to Iso plus IBMX, the dynamic range of all three probes turns out to be very similar: Epac2-camps, 20±1.1%; Epac2-MyrPalm, 17±0.7%; and Epac2-CAAX, 17±1.0%. The fact that MDL did not affect the baseline response of Epac2-camps and Epac2-MyrPalm, indicates that the basal levels of cAMP found where these probes are expressed is below their activation threshold (≤10 nM). Using equation 1(see *Materials and Methods*), the basal concentration of cAMP detected by Epac2-CAAX was estimated to be 93±21 nM (n = 24), or at least 10 fold higher.

**Figure 6:**
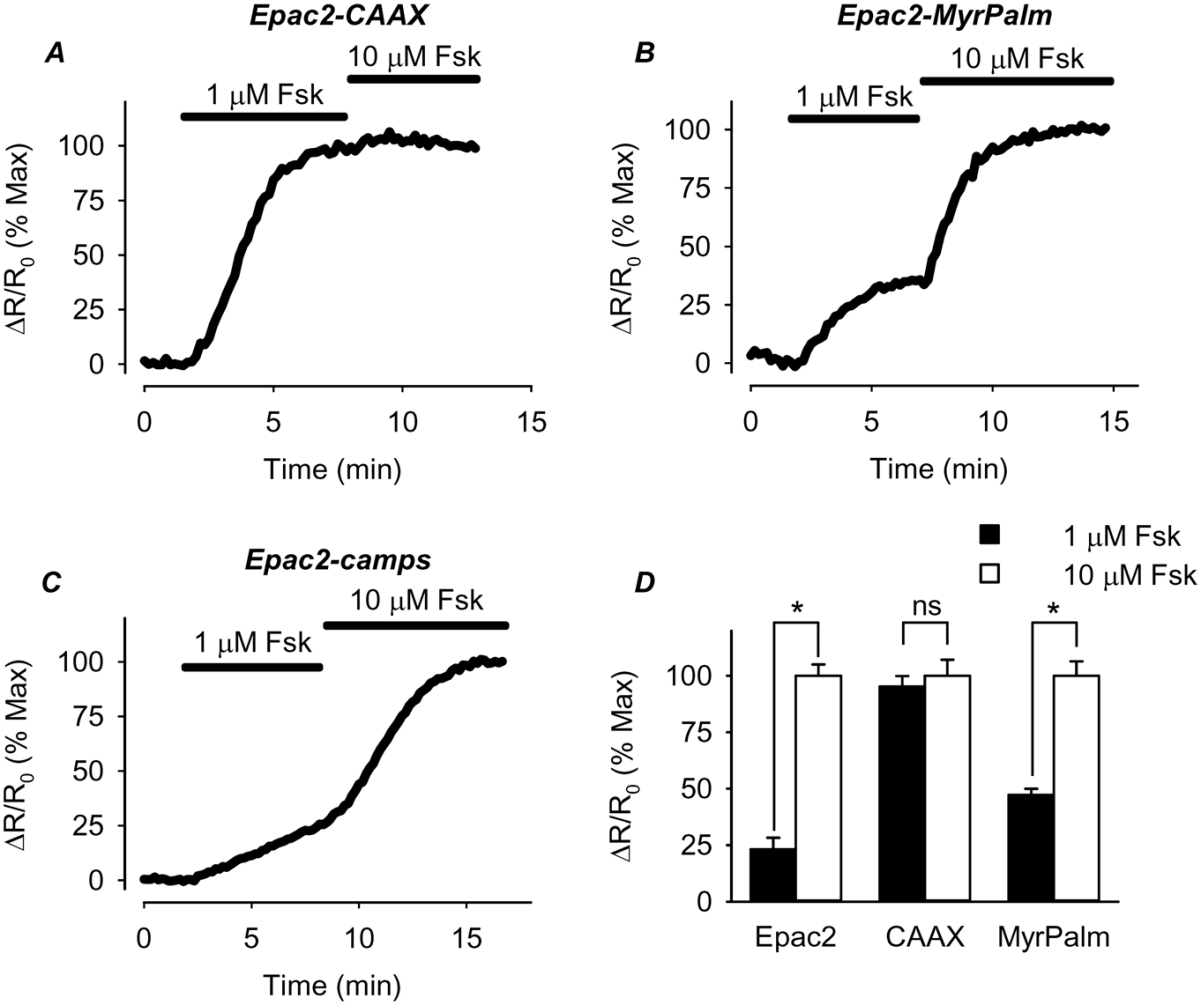
Effect of direct adenylyl cyclase (AC) stimulation on cAMP activity in different microdomains. ***A–C***, time course of changes in normalized FRET response (ΔR/R_0_) in cells expressing Epac2-camps (Epac2), Epac2-CAAX (CAAX), and Epac2-MyrPalm (MyrPalm) following exposure to a sub-maximally stimulating concentration of the AC activator forskolin (Fsk; 1 µM). Responses are normalized to the magnitude of the response produced by subsequent exposure to a maximally stimulating concentration of Fsk (10 µM). ***D***, comparison of average changes in normalized FRET responses (n = 7–12; *p<0.001).

Higher basal levels of cAMP can be explained if cAMP production is elevated and/or cAMP hydrolysis is reduced. The fact that MDL inhibited the baseline response of Epac2-CAAX indicates that there is significant basal AC activity stimulating cAMP production associated with non-lipid raft domains of these cells. To determine whether this basal AC activity is higher than it is in other regions of the cell, we examined the sensitivity of the response of the different probes to forskolin, an agonist that directly activates AC independent of receptor activation (**figure 6**). The prediction is that submaximally stimulating concentrations of forskolin should produce a greater cAMP response in regions where there is a greater amount of basal AC activity. Consistent with this idea, the Epac2-CAAX FRET response produced by 1 µM forskolin was 95±4.6% (n = 11) of the maximum response observed following subsequent exposure to 10 µM forskolin. This was significantly larger than the magnitude of the normalized FRET response produced by Epac2-camps (23±5.1% of max, n = 7) or Epac2-MyrPalm (47±2.7% of maximum, n = 12). These results indicate that the higher basal level of cAMP associated with non-lipid raft domains of the cell is due at least in part to a greater amount of basal AC activity.

**Figure 7:**
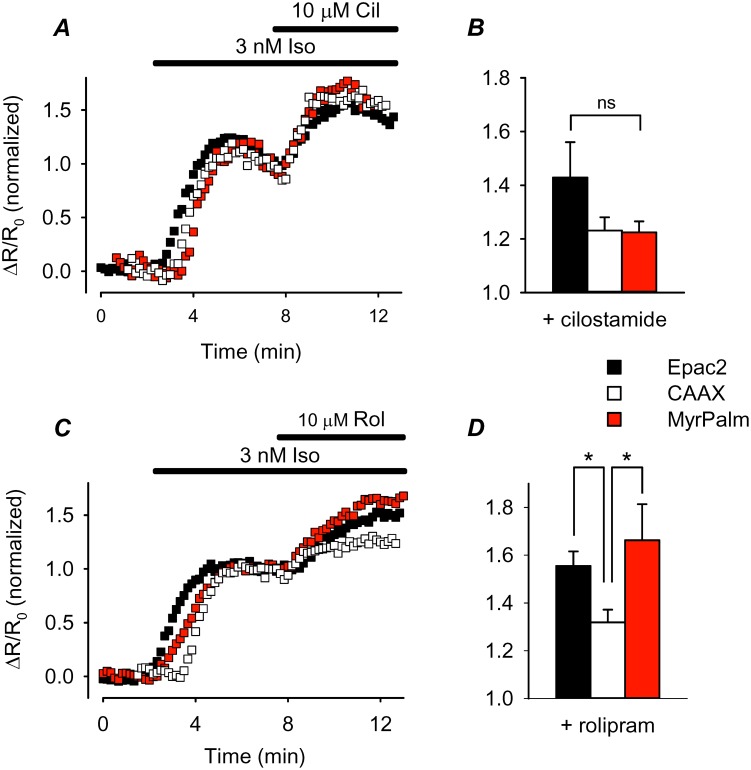
Effect of phosphodiesterase (PDE) inhibition on cAMP activity in different microdomains. ***A*** and ***C***, time course of changes in normalized FRET response (ΔR/R_0_) recorded in cells expressing Epac2-camps (Epac2), Epac2-CAAX (CAAX), and Epac2-MyrPalm (MyrPalm) following exposure to a sub-maximally stimulation concentration of isoproterenol (Iso; 1 nM) and subsequent addition of selective the selective PDE3 inhibitor cilostamine (Cil) or the selective PDE4 inhibitor rolipram (Rol). Responses to PDE inhibition are normalized to the magnitude of the response produced by 1 nM Iso alone. ***B***, comparison of average changes in normalized FRET responses to 10 µM cilostamide (n = 8–13; ns  =  not significant). ***D***, comparison of average changes in normalized FRET responses to inhibition of PDE4 activity with rolipram 10 µM rolipram (n = 5–11; *p<0.05).

A reduction in cAMP metabolism due to lower PDE activity could also contribute to higher basal levels of cAMP. If this is true, then one would expect cAMP responses in those subcellular locations to be less sensitive to PDE inhibition. Although multiple PDE isoforms are capable of metabolizing cAMP, PDE3 and PDE4 are the predominant subtypes in HEK293 cells [44], [45]. We tested the role of each of these PDE isoforms using subtype-selective inhibitors. Exposure to either cilostamide alone (10 µM), a selective PDE3 inhibitor [46], or rolipram alone (10 µM), a selective PDE4 inhibitor [47], did not produce a detectable change in FRET responses by any of the probes (data not shown), suggesting that differences in PDE activity may not contribute significantly to differences in basal cAMP activity. To evaluate the potential role of differences in PDE activity further, we examined the effect of these inhibitors in the presence of a submaximally stimulating concentration of Iso (3 nM). Under these conditions, inhibition of either PDE3 or PDE4 produced cAMP responses that could be detected by all three probes (**figure 7**). Although there was some variability, when normalized to the magnitude of the response produced by 3 nM Iso alone, the effect that inhibition of PDE3 had on the responses detected by Epac2-camps (43±13%; n = 9), Epac2-CAAX (23±5.0%; n = 13), and Epac2-MyrPalm (22±4.1%; n = 8) were not statistically different (**figure 7A**). However, inhibition of PDE4 did have a more significant effect on the normalized responses detected by Epac2-camps (55±6.1%; n = 8) and Epac2-MyrPalm (66±15%; n = 5) than it did on the response of Epac2-CAAX (32±5.3%; n = 11) (**figure 7D**).

### Modeling Compartmentalized cAMP Responses

We further investigated the mechanisms responsible for cAMP compartmentation in HEK293 cells using a computational approach, by constructing a single cell model of cAMP signaling that consists of lipid raft and non-lipid raft sub-plasma membrane domains and a bulk cytosolic domain. The total cell volume was assumed to be 2.5 pL [11], with 5% of that attributed to the membrane associated compartments. Rich et al. [10], [11] previously described cAMP signaling in HEK293 cells using a two compartment (membrane and cytosol) model, where the membrane compartment volume was calculated as 1.5 to 2% of a spherically shaped cell. However, the morphology of HEK cells is flat, in which case a membrane compartment of similar thickness would make up a larger proportion of the total cell volume. Lipid rafts made up 30% of the total membrane compartment volume. Assuming both membrane compartments have similar surface to volume ratios, lipid rafts would then comprise 30% of the cell surface area, which is consistent with experimental estimates of 13 to 50% [20].

**Figure 8:**
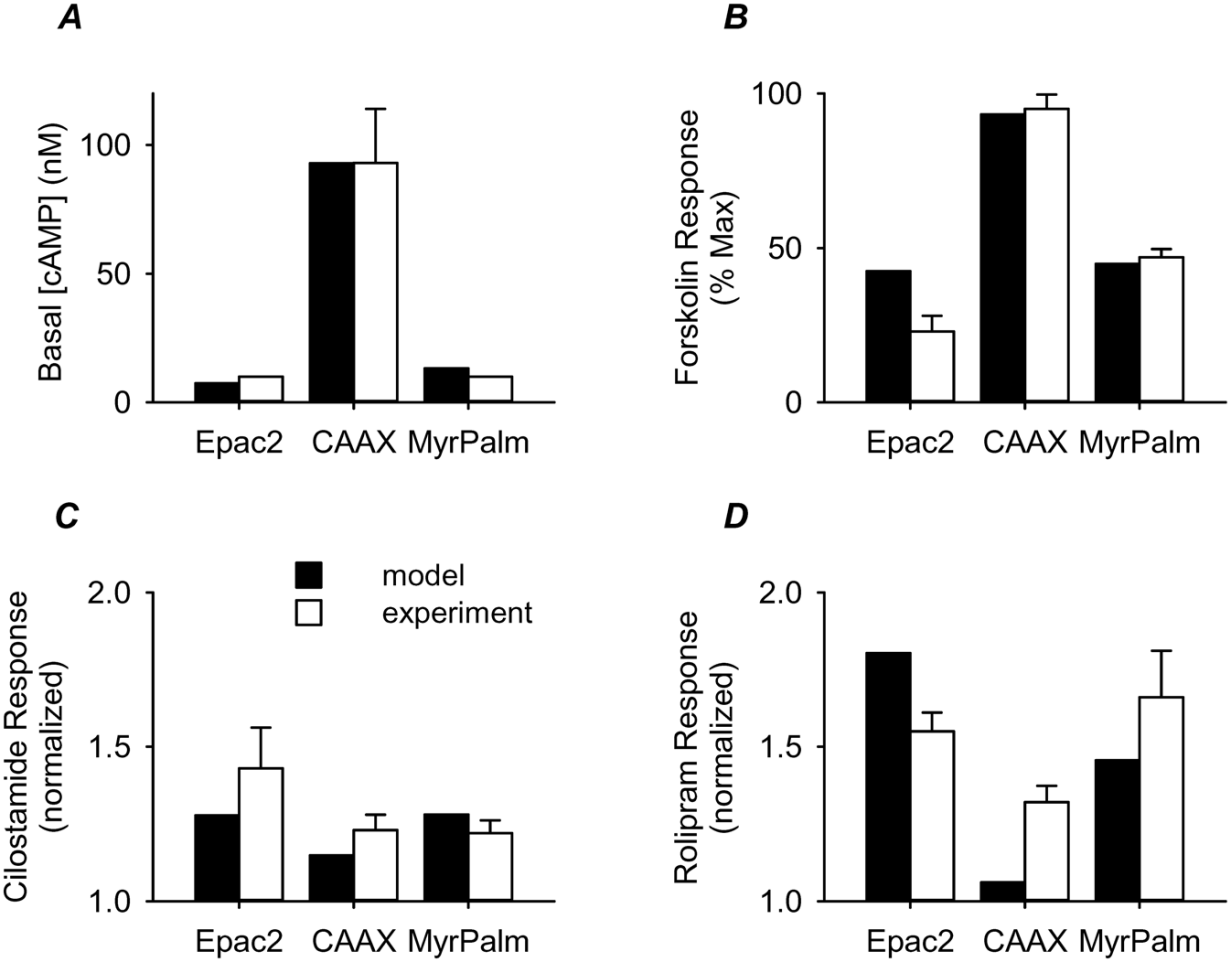
Comparison of computational and experimental results. ***A***, basal cAMP concentration. ***B***, response to a 20 fold increase in adenylyl cyclase (AC) activity (model) and 1 µM forskolin (experiment) normalized to 300 fold increase in AC activity (model) and 10 µM forskolin (experiment). ***C***, response to a 90% reduction of PDE3 activity (model) and 10 µM cilostamide in the presence of 3 nM isoproterenol (experiment) normalized to response to 3 fold increase in basal AC activity (model) and 3 nM isoproterenol (experiment). ***D***, response to a 40% reduction of PDE4 activity (model) and 10 µM rolipram in the presence of 3 nM isoproterenol (experiment) normalized to a 5 fold increase in basal AC activity (model) and 3 nM isoproterenol (experiment).

Initial values for the other model parameters were based on experimental data as described in **table 1**. These parameters were then optimized simultaneously to several of our experimental findings. This included the estimated basal cAMP levels in the three microdomains (**figure 8A**), as well as the effect of receptor independent stimulation of AC activity (**figure 8B**) and inhibition of PDE3 (**figure 8C**) and PDE4 (**figure 8D**) activity. Basal AC activity was assumed to be ∼4% of the maximal receptor stimulated value previously used by Rich et al. [10]. This is in line with the basal rate of production estimated from experimental studies [48]. The maximal rate of cAMP hydrolysis by PDEs was significantly less than the values used in earlier models [10], [14], but much closer to estimates calculated from previously published experimental data [44]. Consistent with the findings of Lynch et al. [44], the model was constrained by assuming that the majority of cAMP hydrolysis was due to PDE4 activity, while PDE3 activity accounted for the remainder. Furthermore, in keeping with the experimental results of Matthiesen and Nielson [45], PDE3 activity was included only in the membrane compartments, while the majority of the PDE4 activity was confined to the bulk cytoplasmic compartment. However, with these constraints on the activity and/or distribution of AC and PDE activity, it was also necessary to assume that the movement of cAMP between compartments was restricted. Using cAMP flux rates between the membrane compartments and the bulk cytoplasmic compartment that are in line with those previously described by Rich et al. [10], [11], the model was able to approximate key experimental findings of the present study (**figure 8**).

**Table 1:** Model parameters.

	Parameter	Initial Value	Units	Constraints	Final Value	Ref
	Total cell volume	2.5	pL	fixed	2.5	[11]
***V_bulk_***	Bulk cytoplasmiccompartment volume	2.375	pL	fixed	2.375	[11]
***V_lipid_***	Lipid raft compartmentvolume	0.0375	pL	fixed	0.0375	[20]
***V_non-lipid_***	Non-lipid raft compartmentvolume	0.0875	pL	fixed	0.0875	[20]
	Total PDE activity	0.35	µM/s	fixed	0.35	[44]
	Total PDE3 activity	0.095	µM/s	<33% of Total PDE activity	0.095	[44]
***PDE3_non-lipid_***	PDE3 V_max_ in non-lipid rafts	0.075	µM/s	<50% of Total PDE3 activity	0.06	[45]
***PDE3_lipid_***	PDE3 V_max_ in lipid rafts	0.02	µM/s	Total PDE3 activity - *PDE3_non-lipid_*	0.035	[45]
***K_mPDE3_***	PDE3 K_M_ for cAMP	0.38	µM	0.18–0.38	0.38	[66]
	Total PDE4 activity	0.255	µM/s	Total PDE activity - Total PDE3 activity	0.255	[44]
***PDE4_non-lipid_***	PDE4 V_max_ in non-lipid rafts	0.028	µM/s	Total PDE4 - *PDE4_bulk_ - PDE4_lipid_*	0.055	[44], [45]
***PDE4_lipid_***	PDE4 V_max_ in lipid rafts	0.027	µM/s	Total PDE4 - *PDE4_bulk_ - PDE4_non-lipid_*	0.01	[44], [45]
***PDE4_bulk_***	PDE4 V_max_ in bulk cytoplasm	0.2	µM/s	>50% of total PDE4 activity	0.19	[45]
***K_mPDE4_***	PDE4 K_M_ for cAMP	1.2	µM	1.2–10	1.23	[66]
	Total AC activity	0.05	µM/s	fixed	0.05	[48]
***AC_lipid_***	AC activity in lipid rafts	0.0015	µM/s	<40% of Total AC activity	0.005	
***AC_non-lipid_***	AC activity in non-lipid rafts	0.0485	µM/s	Total AC activity - *AC_lipid_*	0.045	
***flux1***	Flux rate between*V_non-lipid_* and *V_bulk_*	2.90E-14	Liters/s	model output	2.87E-14	[10], [11]
***flux2***	Flux rate between*V_lipid_* and *V_bulk_*	4.49E-14	Liters/s	model output	4.45E-14	[10], [11]
***flux3***	Flux rate between*V_non-lipid_* and *V_lipid_*	3.68E-15	Liters/s	model output	1.47E-15	[10], [11]

Adenylyl cyclase (AC) and phosphodiesterase (PDE) activities were estimated from published rates of cAMP production or hydrolysis (pmol/mg/min), respectively, assuming HEK293 cell volume of 2.5 pL [11] and a protein concentration of 1.2 ng/cell (R. Ostrom, personal communication).

## Discussion

### Comparison to Previous Work

A variety of biosensors have been used to demonstrate that cAMP signaling is compartmentalized in intact, living cells. However, most studies have focused on the differences between cAMP responses occurring near the plasma membrane and the rest of the cytosolic compartment [8]–[11], [49]. The implied assumption is often that cAMP signaling near the plasma membrane is uniform. However, given the heterogeneous nature of the cell membrane, this seems unlikely. For example, Wachten et al. [28] demonstrated that receptor activation can produce compartmentalized cAMP responses in GH_3_B_6_ pituitary cells that are created by different AC isoforms found in lipid raft domains of the plasma membrane. In the present study, we tested the hypothesis that compartmentation of cAMP signaling may be a consequence of segregation of signaling proteins between lipid raft and non-lipid raft membrane domains.

In the present study, we used FRAP experiments to demonstrate that our Epac2-based probes were expressed in different membrane domains. Following the disruption of lipid rafts by cholesterol depletion, the mobility of only the raft-associated Epac2-MyrPalm probe was significantly reduced (**figure 1**). Similar treatment had little effect on the mobility of Epac2-CAAX, the biosensor that is presumably excluded from lipid rafts. The mechanism for reduced mobility of lipid raft-associated proteins is still unclear. Some studies have reported that the reduction in the mobility of raft-associated protein following cholesterol depletion is due to the changes in their interaction with actin cytoskeleton [50], [51]. This conclusion was supported by the experiments in which raft-associated proteins exhibited higher mobility following actin cytoskeleton disruption [51]. Other studies have suggested that the presence of cholesterol can locally fluidize the otherwise rigid sphingolipid-rich regions of the membrane [52], [53]. Consistent with this mechanism, several studies have demonstrated that cholesterol depletion preferentially reduces the mobility of raft-associated proteins [39], [54], [55].

The idea that the targeting sequences we used are directing our probes specifically to lipid raft and non-lipid raft domains in the plasma membrane is further supported by previous studies, where membrane fractionation experiments were used to show that the addition of MyrPalm leads to co-localization in membrane fractions where raft marker proteins are found, while attachment of the CAAX sequence leads to exclusion from those membrane fractions [31]–[34].

Using an approach similar to the present study, Depry et al. [33] targeted the FRET-based A-kinase activity reporter (AKAR) to different lipid raft and non-lipid raft membrane domains in HEK293 cells. In this complementary study, they found evidence that basal PKA activity associated with lipid rafts is greater than that found in non-lipid rafts. This might seem to contradict our present findings, which indicate that there is greater basal cAMP activity associated with non-lipid raft domains. However, direct comparison of results from the two studies is complicated by differences in the actual biosensors used. Activity of the AKAR probe can be affected by differences in cAMP levels, but because it actually responds to phosphorylation, its activity is also affected by differences in PKA expression levels, as well as differences in phosphatase activity.

### Membrane Domains and Compartmentation of Receptor Mediated Responses

HEK293 cells express both βARs and EPRs. However, membrane fractionation studies have demonstrated that βARs are associated primarily with lipid raft fractions, while EPRs are associated with non-lipid raft fractions [8]. Evidence for differences in the subcellular pattern of cAMP produced by βARs and EPRs was not evident in our initial experiments (compare **figures 2** and **3**), because cAMP produced following maximal receptor activation alone resulted in saturation of all three biosensors used in the present study. This was demonstrated by the fact that subsequent addition of the non-specific PDE inhibitor IBMX did not cause any further increase in the FRET response observed. However, cholesterol depletion did selectively alter responses to βAR stimulation, without affecting PGE1 responses, supporting the idea that these receptors are indeed found in different membrane domains.

Cholesterol depletion also revealed evidence that raft and non-raft microdomains of the plasma membrane contribute to receptor-dependent compartmentation of cAMP production (**figure 4**). Under these conditions, there was a significant decrease in the size of the cAMP response occurring in lipid raft domains, as detected by Epac2-MryPalm. In addition, there was a significant decrease in the size of the response occurring throughout the entire cell, as detected by Epac2-camps, which supports the idea that most of this cAMP originated from βARs found in lipid rafts. βAR stimulation did, however, produce a significant increase in cAMP in non-lipid raft domains, as detected by Epac2-CAAX. Yet this response was not significantly affected by cholesterol depletion. This is consistent with the idea that at least some fraction of the βARs in HEK293 cells are located in non-raft regions of the plasma membrane [8].

Activation of EPRs, like βARs, produced responses that were detected in the bulk cytoplasmic compartment, as well as microdomains associated with lipid rafts and non-lipid rafts. However, the observation that none of these responses were affected by cholesterol depletion indicates that they were mediated by receptors found primarily in non-lipid raft fractions of the plasma membrane. This suggests that in HEK293 cells, cAMP produced by EPRs may not be influenced by the same mechanisms that restrict cAMP signaling by βARs found in non-lipid raft domains. It is possible that not all non-raft domains are identical, and the mechanisms for compartmentation may vary. Alternatively, there may be significantly more EPRs in non-raft domains, and the size of the cAMP response simply overwhelms any mechanism for compartmentation.

The effect that cholesterol depletion has on GPCR responses is quite mixed [42]. It has been reported to both facilitate and inhibit many different receptor mediated responses. The exact explanation for the apparent discrepancies is unclear. In some cases, the variability may be a function of the specific type of receptor and/or cell involved. Consistent with the present study, we previously reported that cholesterol depletion altered βAR, but not EPR, responses in cardiac myocytes [22]. However, in that study, cholesterol depletion actually enhanced the response to βAR stimulation. In cardiac myocytes, βAR stimulation of cAMP production involves the regulation of AC5/6 activity in caveolae, a specific subset of lipid rafts associated with the scaffolding protein caveolin [56]. It has been hypothesized that cholesterol depletion in cardiac cells disrupts an inhibitory interaction between caveolin and AC, resulting in increased cAMP production [22], [57]. However, not all HEK293 cells express endogenous caveolins [57], [58]. This might explain why cholesterol depletion did not enhance βAR production of cAMP associated with lipid rafts in the present study. The decrease in cAMP production may have been due to disruption of receptor coupling with downstream signaling elements [27].

### Membrane Domains and Compartmentation of Basal cAMP

We also observed a difference in the magnitude of the FRET response recorded in different subcellular locations following receptor activation. The maximal response detected by Epac2-camps and Epac2-MyrPalm was significantly greater than that detected by Epac2-CAAX. Previous studies have suggested such findings reflect cAMP compartmentation due to PDE activity [9]. However, exposure to the non-selective PDE inhibitor IBMX did not alter any of the responses produced by maximal βAR or EPR activation in the present study. Instead, we found evidence that the difference in the magnitude of the maximal responses was due to differences in the basal cAMP level in different subcellular locations. Consistent with this conclusion, we found that inhibition of basal AC activity with MDL12330A produced a significant decrease in the FRET response of the Epac2-CAAX probe, but not the other probes. This indicates that not only do membrane microdomains contribute to differences in βAR signaling, but they also are associated with differences in basal cAMP levels. Using the approach previously described [43], we estimated the actual concentrations of cAMP under basal conditions in the different domains. Consistent with previous studies, we found that cAMP levels tend to be higher near the plasma membrane than they are throughout the rest of the cell [9]–[11], [28], [45], [49]. However, in the present study, we demonstrate there are significant differences between the levels associated with lipid-raft and non-lipid raft membrane domains.

The explanation for the difference in basal cAMP levels appears to be due at least in part to differences in basal AC activity. This is supported by the observation that cAMP responses in non-lipid raft domains were more sensitive to direct activation of AC with forskolin (see **figure 6**). It has been shown that different AC isoforms may be targeted to specific membrane domains as determined by membrane fractionation. In HEK293 cells, AC5/6 is concentrated in lipid rafts, while AC2, AC4, and AC9 are found exclusively in non-lipid raft membrane fractions [16]. Furthermore, AC2 has been shown to exhibit a greater intrinsic basal activity than AC5 or AC6 [59], [60]. Differences in forskolin sensitivity could also be explained by enhanced basal interactions with the stimulatory G protein G_s_ as well as intrinsic GPCR activity [61].

We also examined the possibility that differences in basal cAMP activity might be explained by non-uniform distribution of PDEs. Previous studies have focused on PDE4 activity as the predominant isoform in HEK293 cells [10]. However, more recent studies have found that approximately 30% of the PDE activity in HEK cells may be attributed to PDE3 [44], [45]. We evaluated the potential contribution of both isoforms using a pharmacologic approach. Inhibition of either PDE3 or PDE4 activity alone had no effect on basal cAMP activity detected by any of the probes. So the only way we could evaluate the potential effect of PDE activity in the different domains was following partial activation of AC activity with Iso. In this case, inhibition of both isoforms had an effect. However, the only significant difference we saw was a slightly smaller effect of PDE4 inhibition on cAMP responses detected by Epac2-CAAX in the non-lipid raft domain. This suggests that non-uniform distribution of PDE4 activity might conceivably contribute to the variations in basal cAMP activity associated with different membrane domains. However, βAR activation of protein kinase A may also upregulate PDE3 and PDE4 activity through a positive feedback mechanism, which might explain why inhibition of PDE activity had no detectible effect under basal conditions.

### Computational Analysis

Previous studies have provided evidence clearly demonstrating that PDE activity contributes to differences between cAMP levels near the membrane and those in the bulk cytosolic compartment in HEK293 cells [9]–[12]. In fact, Oliveira et al. [14] used a computational approach to conclude that PDE activity alone is sufficient to explain cAMP compartmentation. However, PDE activity was assumed to be exceptionally high in that study. The authors found that compartmentalized behavior was lost when PDE activity was reduced to a level that is still orders of magnitude greater than estimates calculated using the experimental results of Lynch et al. [44]. Based on those estimates, our model supports the idea that PDE activity contributes to compartmentation, our results also indicate that differences in total AC activity play a role. However, the model clearly demonstrates that while these factors are critical, they alone are not sufficient.

Our calculations indicate that compartmentation can only be explained if the diffusion of cAMP between compartments is limited. Without this, all compartmentalized behavior is lost. It is difficult to estimate actual diffusion coefficients from the flux rates used in our model, because this requires information about the size and shape of the microdomains that is currently unknown. Using assumptions similar to those of Rich et al. [11], it is clear that the movement of cAMP throughout the cell must be significantly slower than free diffusion. Our current results suggest, however, that the average rate of cAMP diffusion between membrane domains is slower than the rate of diffusion between either of the membrane domains and the bulk cytoplasmic compartment. At present, we are not able to reasonably model the PGE1 and Iso data because both of those receptors produce saturating responses, limiting our ability to define the cAMP concentrations they produce.

## Conclusions

The results of the present study demonstrate that cAMP signaling near the plasma membrane is not homogeneous. Compartmentation of βAR mediated cAMP responses appears to correlate with the microdomain in which the receptors are expressed. βARs associated with both lipid raft as well as non-lipid raft domains stimulate cAMP production, but only those receptors associated with lipid rafts appear to contribute to global cAMP responses. βARs associated with non-lipid rafts appear to produce more localized cAMP responses. There are also differences in basal cAMP levels associated with lipid raft and non-raft microdomains. Differences in adenylyl cyclase and PDE activity may contribute to some of these behaviors, but quantitative modeling suggests that these factors alone are not sufficient.

## Materials and Methods

### Plasmid Construction

Plasmids were constructed using the pcDNA3.1 (Life Technologies) mammalian expression vector. The Epac2-camps FRET-based cAMP biosensor was modified by site-directed mutagenesis to add targeting sequences in order to record changes in cAMP levels associated with specific membrane microdomains (**figure 1A**). Epac2-MyrPalm was designed to report cAMP changes in membrane raft domains. This plasmid contains an N-terminal acylation sequence (GCINSKRKD) from Lyn kinase, which results in myristoylation and palmitoylation that targets proteins to lipid raft/caveolar membrane fractions [31]. Epac2-CAAX was designed to report cAMP changes in non-raft membrane microdomains. This plasmid contains the CAAX box sequence (KKKKSKTKCVIM) from Rho GTPase attached to carboxyl terminus of Epac2-camps (**figure 1A**), which results in specific targeting of proteins to non-lipid raft domains of the plasma membrane [31]. The DNA fragments encoding Epac2-CAAX and Epac2-MyrPalm were amplified from pcDNA constructs using Platinum PCR Supermix High Fidelity (Agilent Technologies, CA). Amplified sequences were then directionally cloned into pShuttle-CMV vector in MCS using HindIII(5′) and EcoRV(3′) restriction sites. Adenovirus constructs of these plasmids were generated using AdEasy XL adenoviral vector system (Agilent Technologies, CA) following the manufacturer’s protocol.

### Cell Culture

Human embryonic kidney (HEK) 293 cells were maintained in DMEM containing 10% fetal bovine serum as well as 100 units/ml penicillin and 100 µg/ml streptomycin. For FRAP and FRET experiments, cells were grown on 35 mm glass-bottom fluorodishes (World Precision Instruments, Inc.). Cells were transduced with adenoviruses using a multiplicity of infection (MOI) of 1–10 for each virus. Cells were used 48–72 hours post transduction. All experiments were conducted at room temperature unless otherwise stated. In cholesterol depletion experiments, cells were incubated in DMEM containing 5 mM methyl-β-cyclodextrin (MBCD) for 1 hour at 37°C.

### Imaging Experiments

All experiments were conducted using cells bathed in the following solution (in mM): NaCl 137, KCl 5.4, MgCl_2_ 0.5, CaCl_2_ 1.0, NaH_2_PO_4_ 0.33, glucose 5.5, and HEPES 5 (pH 7.4). FRET imaging was conducted on the stage of an inverted microscope (Olympus IX71) using an OrcaD2 dual chip CCD camera and HCImage data acquisition and analysis software (Hamamatsu). Changes in cAMP activity were defined as the change in background and bleed-through corrected eCFP/eYFP fluorescence intensity ratio (ΔR) relative to the baseline ratio (R_0_) measured throughout the entire cell as described previously [22], [30], [62], [63].

Confocal imaging was performed using an Olympus Fluoview 1000 confocal microscope. The expression pattern of the various biosensors was determined using an argon laser (515 nm line) to excite eYFP. Images were exported as tiff files, and the contrast and brightness of these images were adjusted in ImageJ software for presentation purposes. Fluorescence recovery after photobleaching (FRAP) experiments were conducted using Olympus Diffusion Measurement Package (DMP) software. The diameter of a 2.5 µM circular region was aligned with the membrane along the edge of the cell. This area was then bleached using the laser at full (100%) power. Images were collected every 1.5 s before and after bleaching using a laser intensity of 1–2% (**figure S1**). Fluorescence recovery curves were generated by plotting recovery of relative fluorescence in the bleached area as a function of time. The mobile fraction (M_f_) and fluorescence recovery half time (t_1/2_) were calculated as described by DiBenedetto et al. [64], using the formula: *M_f_  =  (F_∞_ – F_0_)/(F_i_ – F_0_)* where *F_∞_* is the fluorescence in the bleached area at the end of the recovery period, F_0_ is fluorescence just after photobleaching, and *F_i_* is the fluorescence before bleaching. The t_1/2_was calculated as the time required for the fluorescence intensity to recover to 50% of *F_∞_*. All measurements were corrected for background fluorescence and any photobleaching that may have occurred during image acquisition.

### Calibration of Biosensors

Lysates of HEK293 cells expressing each cAMP biosensor were prepared following the method as described by Wachten et al. [28]. Briefly, cells were washed twice with ice-cold phosphate-buffered saline and then centrifuged at 200 g for 5 minutes. The pellet was resuspended in 5 mM Tris-HCl, 2 mM EDTA (pH 7.4) and lysed by passaging through a 21-gauge needle. The lysate was then centrifuged at 200,000 g for 20 minutes at 4°C. FRET measurements were performed in 96 well plates using a Chameleon V multitechnology plate reader (Hidex). Excitation of eCFP was achieved using a 440/20 excitation filter. Emissions of eCFP and eYFP were measured using 480/30 and 535/24 band-pass filters, respectively. The cAMP concentrations producing half-maximal responses (*EC_50_*) and the Hill coefficients (*n*) of the relationships (**figure S2**) were determined by fitting the data to a three parameter logistic equation using SigmPlot (Systat Software).

### Calculation of Cellular cAMP Concentration

The concentrations of cAMP detected *in situ* were estimated using the method according to Borner et al. [43], using the equation:
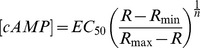
(1)where *EC_50_* and *n* were determined as described above. We previously demonstrated that these values are not significantly affected when measured *in vitro* [30]. The same is not true for the minimum and maximum FRET ratios (*R*), which must be measured in the intact cell [43]. Therefore, *R_min_* was defined as the FRET ratio observed *in vivo* following inhibition of basal adenylyl cyclase activity with 100 µM MDL, and *R_max_* was defined as the FRET ratio observed following exposure to agonist plus IBMX.

### Materials

MDL 12330A, cilostamide, and rolipram were obtained from Tocris. DMEM, penicillin/streptomycin, fetal bovine serum were purchased from Life Technologies. All other reagents were purchased from Sigma-Aldrich unless otherwise stated.

### Computational Modeling

A quantitative model of cAMP signaling consisting of non-lipid raft, lipid raft, and bulk cytosolic compartments was generated using the following equations:

*Non-lipid raft domain*

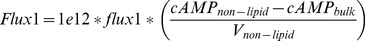
(2)

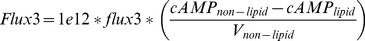
(3)

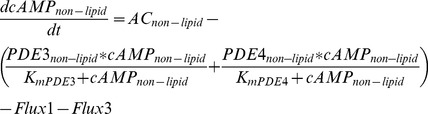
(4)


*Lipid-raft domain*

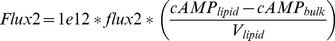
(5)

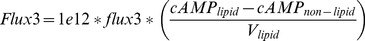
(6)

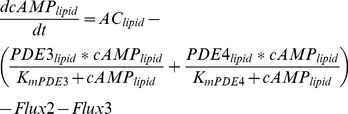
(7)


*Bulk cytosolic domain*

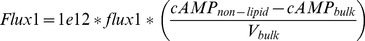
(8)

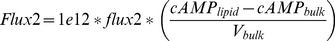
(9)

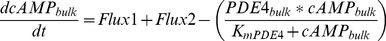
(10)


### Model Construction and Optimization

Definitions and initial values for model parameters were based on experimental data as described in **table 1**. A modified Nelder Mead Simplex constrained optimization method implemented in C++ was used for simultaneous optimization of parameters to four experimental protocols: basal cAMP concentrations in different domains (**figure 8A**), effect of stimulating AC activity with forskolin on FRET responses in different domains (**figure 8B** **)**, comparing effect of PDE3 inhibition on FRET responses in different domains after stimulating AC activity (**figure 8C** **)**, effect of inhibiting PDE4 activity on FRET responses in different domains after stimulating AC activity (**figure 8D**). The optimization determines the set of model parameters that can best simulate the experimental results.

A cost function for each protocol was defined as the sum of squared relative differences between experiment and simulation. The total cost function (sum of the individual protocol errors) was then minimized and converged with a tolerance of 1E-6. During optimization, parameters were allowed to fluctuate within the experimentally reported ranges as indicated in **table 1**.

To simulate the forskolin experiments in **figure 8**, cAMP concentrations were computed under (A) baseline conditions, (B) following 20-fold increased AC activity, and (C) under maximal stimulation (300-fold increase) of AC activity. Using the FRET probe calibration curve, the cAMP concentrations were converted to FRET response values. Finally, we calculated the relative size of the response to partial AC stimulation as a fraction of the response to maximal AC stimulation: (B-A)/(C-A).

For PDE3 and PDE4 inhibition simulations, the model was used to calculate cAMP concentration under (A) baseline conditions, (B) following 3-fold increased AC activity, and (C) following elimination of 90% PDE3 or 40% PDE4 activity. Again, using the FRET probe calibration curve, the cAMP concentrations were converted to FRET response values. Then, we determined the size of the response to elimination of PDE activity relative to the size of the response to partial AC stimulation: (C-A)/(B-A).

We undertook a sensitivity analysis [65] to determine how changes to individual model parameters would affect cAMP FRET probe responses in each of the three subcellular compartments. In each case, the model parameters were increased and decreased by 10%. The relative changes in the predicted responses of each FRET probe were then used to calculate the sensitivity according to the following equation:
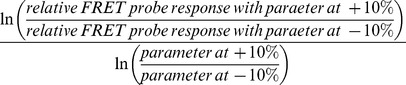
(11)


Results of the sensitivity analysis are illustrated in **figures S3–S5**.

### Statistics

All data are expressed as the mean ? S.E.M of the indicated number of independent experiments (*n*). Statistical significance (P<0.05) was determined by Student’s t-test or one way ANOVA, with Dunnett’s post-hoc analysis where appropriate.

## Supporting Information

Figure S1 Effect of cholesterol depletion on the mobility of the membrane bound biosensors Epac2-CAAX or Epac2-MyrPalm was determined by conducting fluorescence recovery after photobleaching (FRAP) experiments in control and MBCD-treated HEK293 cells. The diameter (2.5 µm) of a circular area was centered over a region of the cell membrane (arrows) and bleached using the 515 nm line of an argon laser at full power. The representative images illustrate the fluorescence intensity before photobleaching (prebleach), immediately after bleaching (bleach), and then 10 and 90 s into the recovery phase.
(TIF)

Figure S2 *In vitro* **c**oncentration-response curves for cAMP activation of FRET based biosensors (*n* = 3–7). *EC_50_*: Epac2-camps, 0.31 µM, Epac2-MyrPalm, 0.43 µM; Epac2-CAAX, 0.16 µM. *Hill coefficient*, Epac2-camps, 0.84; Epac2-MyrPalm, 0.82, Epac2-CAAX, 1.1. See *Materials and Methods* for details.
(TIF)

Figure S3 Sensitivity analysis of parameters used in simulating responses to direct activation of adenylyl cyclase with forskolin. See *Materials and Methods* for details.
(TIF)

Figure S4 Sensitivity analysis of parameters used in simulating responses to inhibition of PDE3 activity with cilostamide. See *Materials and Methods* for details.
(TIF)

Figure S5 Sensitivity analysis of parameters used in simulating responses to inhibition of PDE4 activity with rolipram. See *Materials and Methods* for details.
(TIF)
