# Supplementary figures and images for: Role of Membrane Microdomains in Compartmentation of cAMP Signaling
Source: PLoS One. 2014 Apr 21;9(4):e95835. doi: 10.1371/journal.pone.0095835 (PMC3994114; doi:10.1371/journal.pone.0095835)

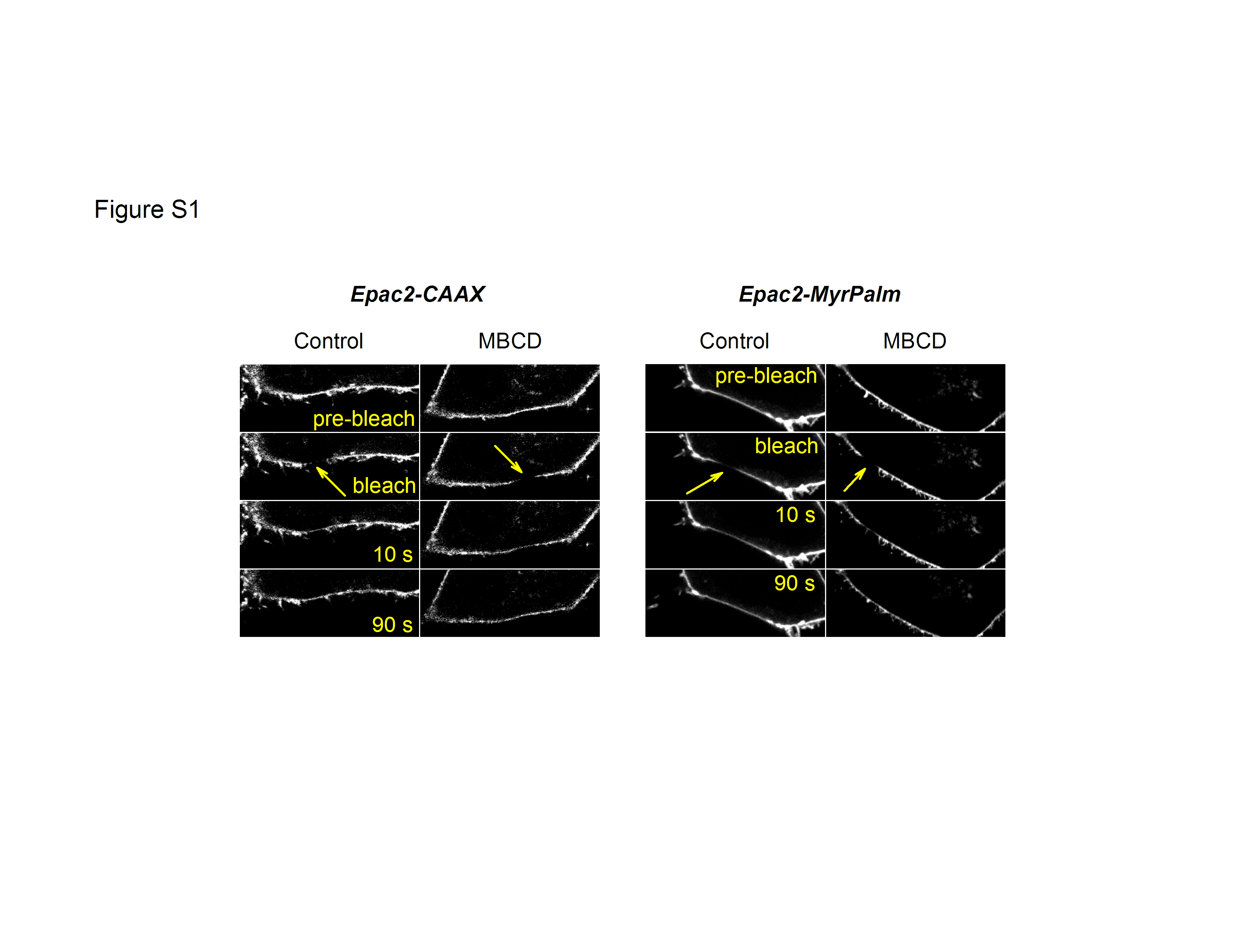

Supplement: Figure S1 — Effect of cholesterol depletion on the mobility of the membrane bound biosensors Epac2-CAAX or Epac2-MyrPalm was determined by conducting fluorescence recovery after photobleaching (FRAP) experiments in control and MBCD-treated HEK293 cells. The diameter (2.5 µm) of a circular area was centered over a region of the cell membrane (arrows) and bleached using the 515 nm line of an argon laser at full power. The representative images illustrate the fluorescence intensity before photobleaching (prebleach), immediately after bleaching (bleach), and then 10 and 90 s into the recovery phase. (TIF) [file pone.0095835.s001.tif]

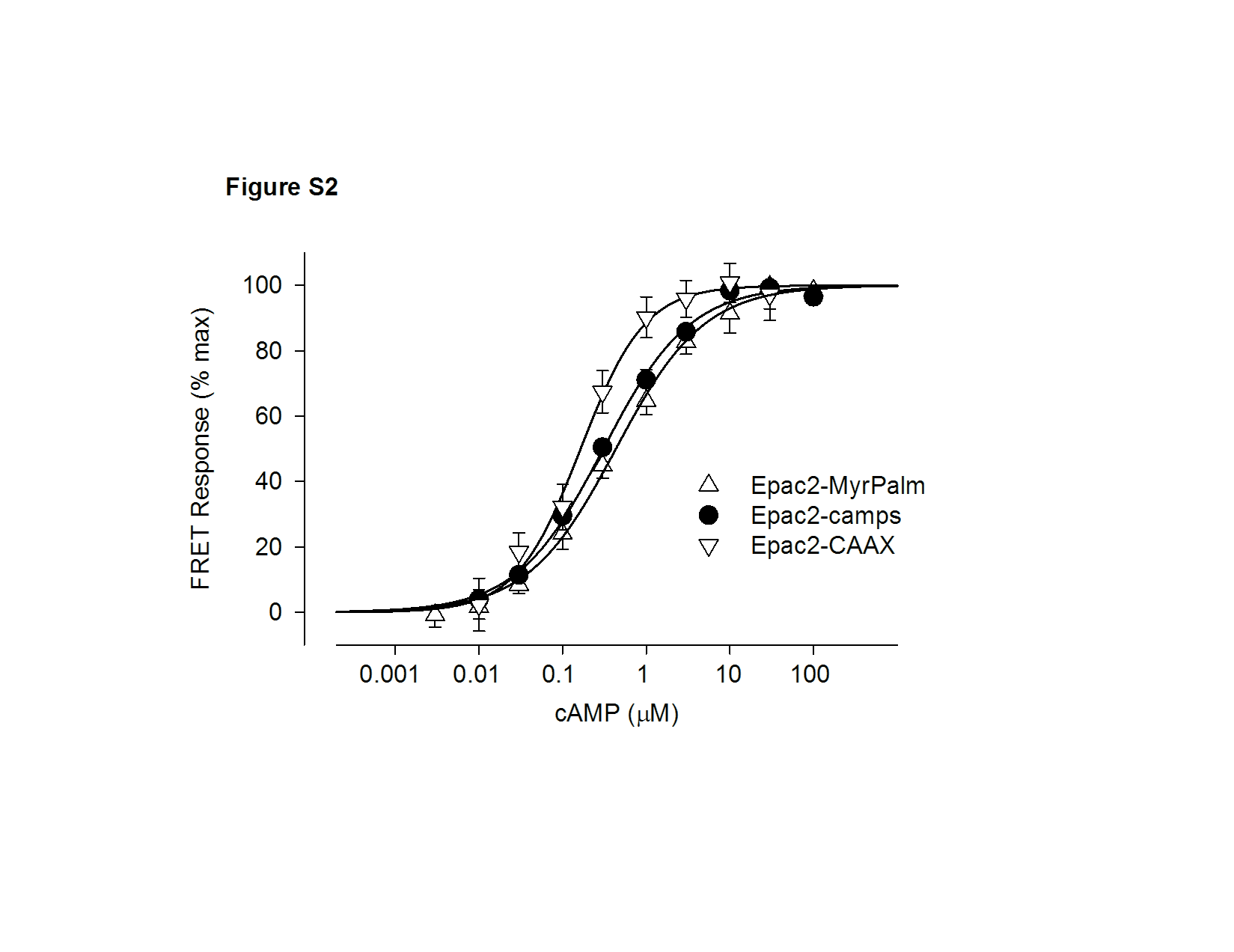

Supplement: Figure S2 — In vitro concentration-response curves for cAMP activation of FRET based biosensors (n = 3–7). EC50: Epac2-camps, 0.31 µM, Epac2-MyrPalm, 0.43 µM; Epac2-CAAX, 0.16 µM. Hill coefficient, Epac2-camps, 0.84; Epac2-MyrPalm, 0.82, Epac2-CAAX, 1.1. See Materials and Methods for details. (TIF) [file pone.0095835.s002.tif]

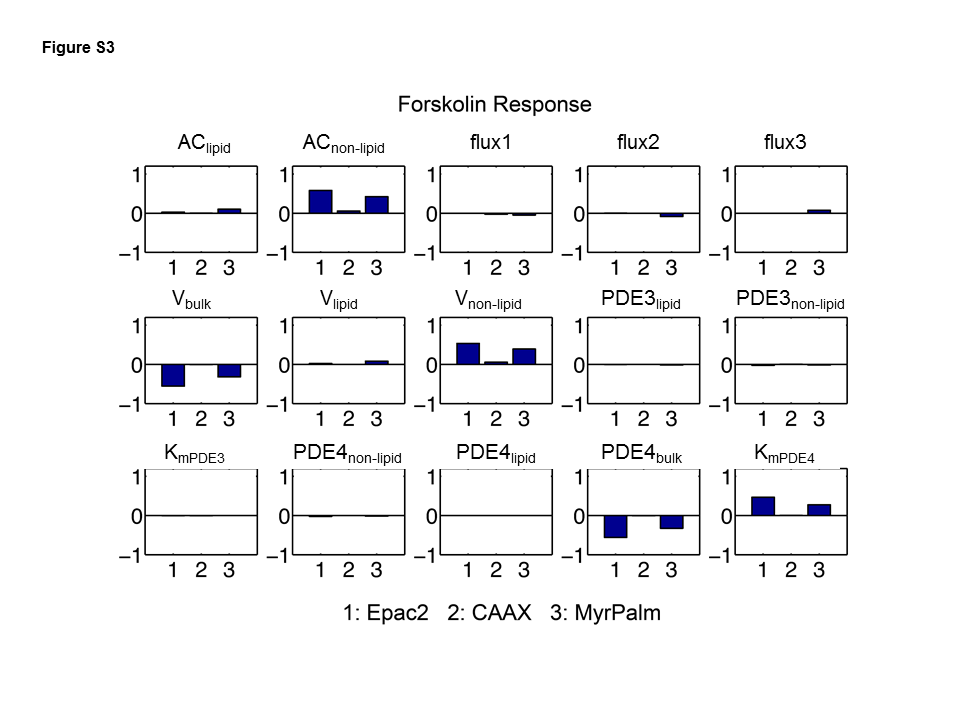

Supplement: Figure S3 — Sensitivity analysis of parameters used in simulating responses to direct activation of adenylyl cyclase with forskolin. See Materials and Methods for details. (TIF) [file pone.0095835.s003.tif]

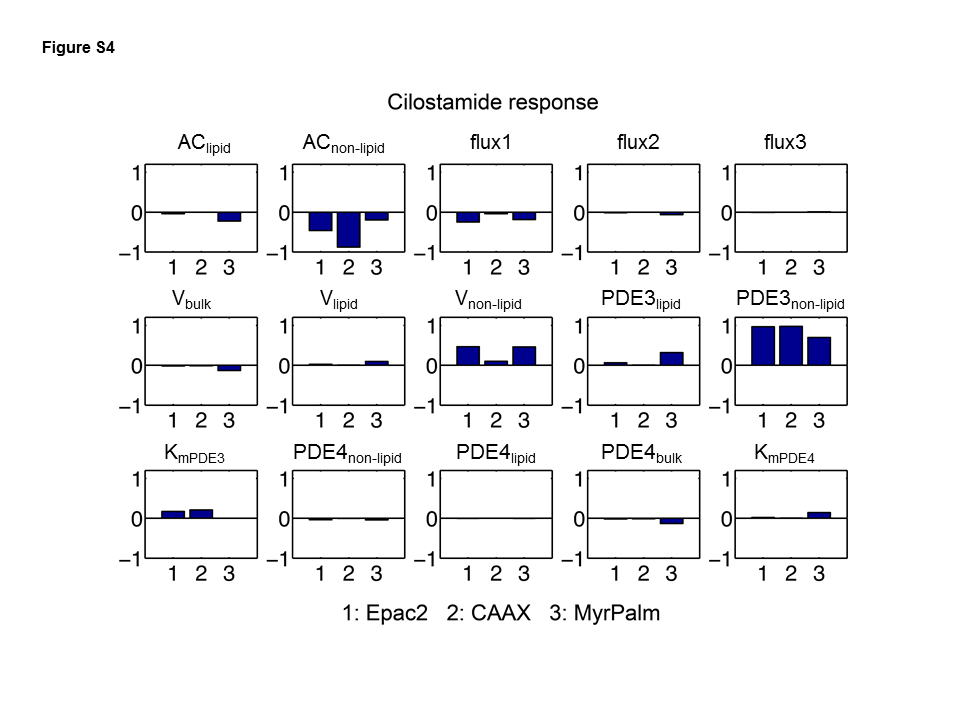

Supplement: Figure S4 — Sensitivity analysis of parameters used in simulating responses to inhibition of PDE3 activity with cilostamide. See Materials and Methods for details. (TIF) [file pone.0095835.s004.tif]

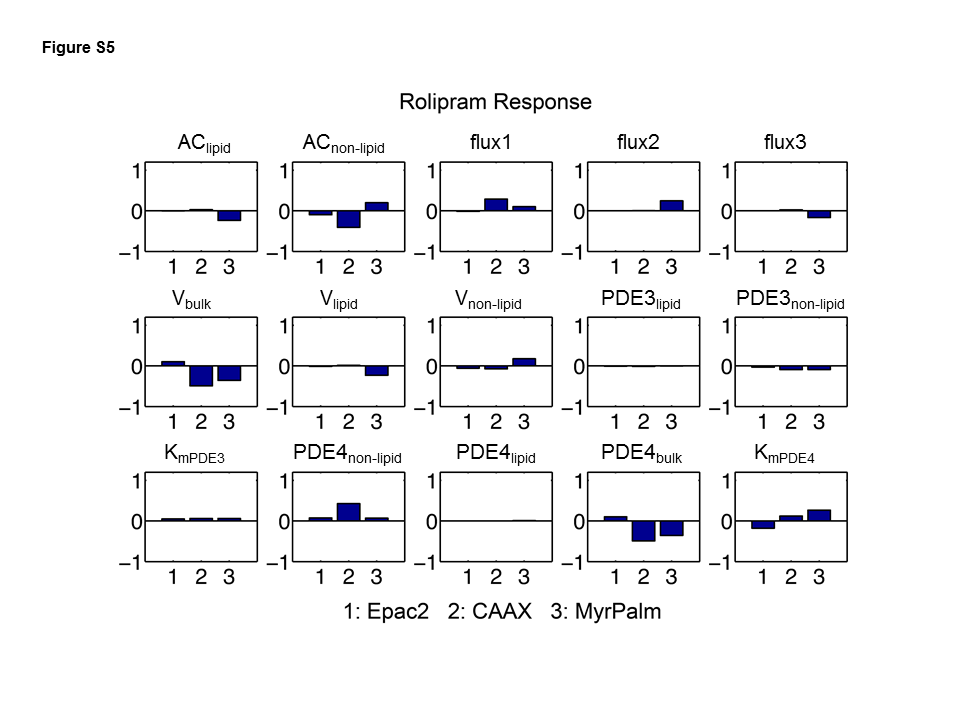

Supplement: Figure S5 — Sensitivity analysis of parameters used in simulating responses to inhibition of PDE4 activity with rolipram. See Materials and Methods for details. (TIF) [file pone.0095835.s005.tif]
